# Long-term psychiatric disorders after traumatic brain injuries in geriatric patients

**DOI:** 10.1192/j.eurpsy.2026.11901

**Published:** 2026-07-31

**Authors:** N. Syrmos

**Affiliations:** Aristotle University, Thessaloniki, Greece

## Abstract

**Introduction:**

Introduction- After traumatic brain injuries, the most disabling problems are generally related to neuropsychiatric results, such as personality change and cognitive impairment.

**Objectives:**

Aim-Aim of this study was to present cases of long-term psychiatric disorders after traumatic brain injuries in geriatric patients.

**Methods:**

Material and Methods-10 geriatrc patients are presented. 9 males(90%) and 1 female(10%). 6 of them present cognitive impairment and 4 of them personality change. Cognitive impairment main characteristic is impaired speed of information processing, poor memory and executive problems. Personality change include poor motivation, and a tendency to be selfcentred and less aware of the needs of others.

**Results:**

Results-Good results in 8 cases (90%) and moderate in 2 (20%). Collaboration between various medical disciplines is essential in order to achive optimal results and clarify the theory,the etiology, and treatment effectiveness.

**Conclusions:**

Conclusions-Recommendations regarding effective psychotherapy, neurological,psychiatric and pharmacotherapy, emerging treatment, and accurate management are necessary in order to achieve optimal overall treatment.Accurate rehabilitation pr minimizes the risk of psychiatric sequelae, but specific psychological and pharmacological treatments may be needed.

**Disclosure of Interest:**

None Declared

